# Supplementary material for: A Likelihood Approach for Real-Time Calibration of Stochastic Compartmental Epidemic Models
Source: PLoS Comput Biol. 2017 Jan 17;13(1):e1005257. doi: 10.1371/journal.pcbi.1005257 (PMC5240920; doi:10.1371/journal.pcbi.1005257)
Supplement: S1 File — (TAR.GZ) [file pcbi.1005257.s014.tar.gz › HSPH_Online-SI-Revision/Additions/MSS11/n100-extreme_noisy/n100-extreme_noisy_NEW2_table-4.pdf]

1	newly 3 weeks spec	10775.3	12839.	{12596., 13055.}	19.152	0.0760591	newly 3 weeks cumul	25108.6	30070.5	{29487., 30671.}	19.7619	0.0782743	newly 5 weeks spec	10934.9	10964.1	{10643., 11234.}	1.78615	0.00774987	newly 5 weeks cumul	47514.6	53858.4	{53326., 54338.}	13.3512	0.0544128	newly 58 weeks cumul	88201.8	81548.1	{79479., 83033.}	7.54372	0.0341486
2	newly 3 weeks spec	8383.	7580.76	{7265., 7787.}	10.2283	0.0467825	newly 3 weeks cumul	21608.	20121.8	{19462., 20537.}	7.52208	0.0338732	newly 5 weeks spec	8027.	6518.13	{6256., 6703.}	19.3583	0.0934103	newly 5 weeks cumul	38163.	34024.	{33028., 34547.}	11.4823	0.0528219	newly 54 weeks cumul	72415.	53052.1	{51883., 53128.}	27.0008	0.13696
3	newly 3 weeks spec	12915.	12342.9	{9680., 13923.}	12.4573	0.0586173	newly 3 weeks cumul	29820.	28573.2	{24027., 31380.}	9.13437	0.0425188	newly 5 weeks spec	11421.	9806.15	{6443., 11838.}	17.082	0.0940312	newly 5 weeks cumul	54424.	50425.1	{39377., 56893.}	12.0769	0.0590517	newly 59 weeks cumul	84212.	71413.9	{49821., 84163.}	15.2164	0.0842018
4	newly 3 weeks spec	15590.	17043.8	{15594., 18250.}	9.87903	0.0404633	newly 3 weeks cumul	27674.	29866.7	{26641., 32758.}	9.4875	0.0388852	newly 5 weeks spec	15345.	15461.6	{14860., 16098.}	2.75653	0.0118751	newly 5 weeks cumul	61225.	64619.8	{60990., 67296.}	5.98233	0.0250661	newly 63 weeks cumul	88766.	89929.6	{81742., 92524.}	4.80126	0.0209067
5	newly 3 weeks spec	10521.	11462.1	{8537., 12952.}	16.6941	0.0697808	newly 3 weeks cumul	21016.	22929.3	{17713., 26012.}	15.6149	0.0646708	newly 5 weeks spec	12782.	12423.7	{8803., 13692.}	11.2141	0.0539289	newly 5 weeks cumul	46785.	48741.	{36423., 54290.}	13.6897	0.0597889	newly 60 weeks cumul	85329.	78964.3	{53409., 90153.}	11.6302	0.0595933
6	newly 3 weeks spec	10702.	11262.9	{10810., 12243.}	7.94253	0.0349465	newly 3 weeks cumul	23963.	24949.3	{23718., 27630.}	6.45645	0.027702	newly 5 weeks spec	11271.	11177.2	{11199., 11559.}	3.33041	0.0196628	newly 5 weeks cumul	47247.	48373.	{47407., 51973.}	5.23752	0.0242624	newly 58 weeks cumul	86171.	78376.9	{78118., 82730.}	9.25137	0.0463256
7	newly 3 weeks spec	11555.	10342.	{9359., 10739.}	10.4973	0.0487777	newly 3 weeks cumul	26529.	24316.4	{21499., 25424.}	8.34046	0.0387048	newly 5 weeks spec	11237.	10353.5	{9646., 10609.}	7.86277	0.0357877	newly 5 weeks cumul	49918.	45703.3	{41515., 47252.}	8.44335	0.0388171	newly 56 weeks cumul	84780.	83294.3	{71405., 86774.}	4.95584	0.022623
8	newly 3 weeks spec	11018.	10915.9	{10853., 11548.}	5.29416	0.027607	newly 3 weeks cumul	24447.	24033.1	{23697., 25331.}	4.50141	0.0226541	newly 5 weeks spec	11207.	10933.9	{11105., 11592.}	5.50906	0.0341069	newly 5 weeks cumul	47781.	46905.8	{47031., 49218.}	4.60687	0.0252433	newly 58 weeks cumul	87124.	75937.2	{78010., 78838.}	12.8851	0.0684378
9	newly 3 weeks spec	10870.	9421.02	{8732., 9728.}	13.3301	0.0624983	newly 3 weeks cumul	22125.	19290.	{17655., 19942.}	12.8138	0.0600159	newly 5 weeks spec	12694.	12042.7	{11786., 12223.}	5.13053	0.0229523	newly 5 weeks cumul	47679.	42974.	{40616., 43902.}	9.86816	0.0453108	newly 58 weeks cumul	88989.	87356.4	{82521., 88690.}	1.83459	0.00828188
10	newly 3 weeks spec	15442.	16264.6	{14413., 17221.}	8.15153	0.0343495	newly 3 weeks cumul	29673.	31022.	{26876., 33286.}	8.61184	0.0365221	newly 5 weeks spec	11765.	13958.	{13439., 14688.}	18.6396	0.0739624	newly 5 weeks cumul	57244.	62420.5	{56867., 64909.}	9.55347	0.039296	newly 62 weeks cumul	72720.	86102.1	{77228., 90360.}	18.4022	0.0726952
11	newly 3 weeks spec	9197.	9209.46	{8723., 9914.}	3.85713	0.0164357	newly 3 weeks cumul	23145.	23850.	{22893., 24636.}	3.47315	0.0145388	newly 5 weeks spec	8893.	7688.67	{7159., 9372.}	15.6243	0.0739512	newly 5 weeks cumul	41559.	40466.	{38839., 44004.}	5.69174	0.0248665	newly 56 weeks cumul	75767.	61119.3	{57188., 76778.}	21.4919	0.105547
12	newly 3 weeks spec	11654.	9056.82	{8555., 9305.}	22.2857	0.109709	newly 3 weeks cumul	24296.	19157.3	{17875., 19737.}	21.1504	0.103465	newly 5 weeks spec	12745.	11353.7	{11206., 11540.}	10.9162	0.0502284	newly 5 weeks cumul	50388.	41474.8	{39699., 42258.}	17.6891	0.0846492	newly 57 weeks cumul	88506.	86624.3	{85063., 86962.}	2.27316	0.00999243
13	newly 3 weeks spec	8371.	9948.6	{9769., 10139.}	18.846	0.0749432	newly 3 weeks cumul	19985.	23881.9	{23616., 24153.}	19.4991	0.0773428	newly 5 weeks spec	8948.	10030.3	{9908., 10152.}	12.095	0.0495599	newly 5 weeks cumul	38014.	44486.3	{44106., 44845.}	17.026	0.0682724	newly 55 weeks cumul	83218.	84797.4	{84603., 84957.}	1.89795	0.00816483
14	newly 3 weeks spec	11434.	5504.08	{4009., 6539.}	51.8622	0.326158	newly 3 weeks cumul	18644.	9828.96	{7345., 11769.}	47.2808	0.286866	newly 5 weeks spec	15310.	11598.	{9847., 12721.}	24.2453	0.123411	newly 5 weeks cumul	50732.	30273.1	{23631., 34550.}	40.3273	0.229543	newly 60 weeks cumul	70240.	86248.7	{77223., 92716.}	22.7915	0.0883906
15	newly 3 weeks spec	13412.	12041.6	{11258., 12948.}	10.2179	0.0473742	newly 3 weeks cumul	27516.	24759.4	{23427., 25858.}	10.018	0.046171	newly 5 weeks spec	12056.	11696.2	{10526., 13524.}	10.9108	0.0482228	newly 5 weeks cumul	54113.	49792.6	{46701., 54078.}	8.09998	0.0374029	newly 60 weeks cumul	75951.	74166.	{66647., 87937.}	13.0192	0.0570073
16	newly 3 weeks spec	15726.	11502.3	{10484., 12298.}	26.8579	0.137571	newly 3 weeks cumul	30526.	22591.8	{19878., 24153.}	25.9915	0.132281	newly 5 weeks spec	14011.	13208.4	{13033., 13805.}	5.72814	0.0279109	newly 5 weeks cumul	61514.	49650.	{47305., 52211.}	19.2867	0.0946628	newly 60 weeks cumul	88752.	84107.5	{81572., 90279.}	6.65713	0.033053
17	newly 3 weeks spec	9053.	9079.23	{8263., 10075.}	7.23705	0.031212	newly 3 weeks cumul	21971.	22075.2	{20475., 24104.}	6.11324	0.0263031	newly 5 weeks spec	9419.	9020.7	{8009., 10129.}	9.14428	0.0413002	newly 5 weeks cumul	40887.	40651.9	{36979., 44816.}	7.10715	0.0309249	newly 55 weeks cumul	79924.	72980.3	{63226., 84870.}	12.9605	0.0608325
18	newly 3 weeks spec	8959.	8724.92	{8449., 8970.}	3.74819	0.0164994	newly 3 weeks cumul	19558.	19404.	{18844., 19966.}	2.70319	0.0116659	newly 5 weeks spec	10392.	10138.3	{9863., 10372.}	2.89944	0.0127984	newly 5 weeks cumul	40376.	39625.4	{38606., 40471.}	2.90158	0.0126965	newly 56 weeks cumul	75084.	75809.6	{74565., 75412.}	1.45155	0.00593648
19	newly 3 weeks spec	11412.	9712.08	{9401., 10043.}	14.8959	0.0702212	newly 3 weeks cumul	23492.	20288.6	{19411., 21477.}	13.636	0.0640388	newly 5 weeks spec	12862.	11618.6	{10915., 12238.}	9.66724	0.0446625	newly 5 weeks cumul	49674.	43514.	{42800., 44191.}	12.4009	0.0575473	newly 58 weeks cumul	89565.	81801.9	{73137., 88662.}	8.66762	0.0412401
20	newly 3 weeks spec	15824.	1367.5	{4., 4353.}	91.3581	1.91526	newly 3 weeks cumul	21990.	2184.59	{11., 6878.}	90.0655	1.7505	newly 5 weeks spec	20039.	4188.39	{12., 13606.}	79.0988	1.53596	newly 5 weeks cumul	66304.	9058.41	{25., 28563.}	86.3381	1.67014	newly 63 weeks cumul	91232.	71787.5	{36641., 91965.}	21.8073	0.28817
21	newly 3 weeks spec	11565.	12117.1	{11146., 13021.}	8.55374	0.0367776	newly 3 weeks cumul	23774.	24619.8	{22805., 26267.}	7.15896	0.0311758	newly 5 weeks spec	12361.	12323.3	{10424., 13617.}	11.5847	0.0519786	newly 5 weeks cumul	49979.	50646.3	{46591., 54433.}	7.67504	0.0339457	newly 60 weeks cumul	80337.	79065.9	{66725., 88004.}	12.9039	0.0589472
22	newly 3 weeks spec	13043.	13184.	{10549., 14790.}	12.3636	0.0546956	newly 3 weeks cumul	25673.	26135.2	{21131., 28977.}	10.7848	0.0472012	newly 5 weeks spec	13126.	12370.4	{9302., 14086.}	13.392	0.0650164	newly 5 weeks cumul	53143.	53042.7	{42486., 59034.}	12.2325	0.0548285	newly 61 weeks cumul	81637.	77883.4	{56981., 91283.}	15.5936	0.0747061
23	newly 3 weeks spec	8971.	9931.35	{9713., 10164.}	10.705	0.0441036	newly 3 weeks cumul	22691.	25244.4	{24788., 25721.}	11.2531	0.0462717	newly 5 weeks spec	8717.	9110.63	{8947., 9263.}	4.51566	0.0191381	newly 5 weeks cumul	40575.	44383.5	{43825., 44948.}	9.38627	0.0389391	newly 54 weeks cumul	74886.	76280.6	{75580., 78174.}	1.86227	0.00796444
24	newly 3 weeks spec	9753.	8426.99	{7743., 8880.}	13.5959	0.0640359	newly 3 weeks cumul	22644.	19956.5	{18063., 21110.}	11.8684	0.0557583	newly 5 weeks spec	10377.	9222.25	{9015., 9403.}	11.128	0.0512852	newly 5 weeks cumul	43672.	38495.8	{36180., 39993.}	11.8525	0.0551168	newly 54 weeks cumul	86096.	75133.	{74437., 76251.}	12.7335	0.059171
25	newly 3 weeks spec	14475.	8361.44	{7285., 8769.}	42.2353	0.239418	newly 3 weeks cumul	28620.	17445.9	{15009., 18458.}	39.0431	0.216283	newly 5 weeks spec	13481.	11074.	{10480., 11352.}	17.8551	0.0856407	newly 5 weeks cumul	57956.	38912.7	{35219., 40494.}	32.8582	0.17366	newly 57 weeks cumul	86463.	87436.4	{74780., 90361.}	4.83336	0.0212455
26	newly 3 weeks spec	8715.	9494.75	{8396., 10220.}	10.7501	0.0439098	newly 3 weeks cumul	21621.	23578.9	{20714., 25480.}	11.1132	0.0453199	newly 5 weeks spec	8698.	8791.49	{8084., 9108.}	4.11784	0.017868	newly 5 weeks cumul	39423.	42060.	{37436., 44675.}	8.89701	0.0369496	newly 55 weeks cumul	81661.	69729.	{63648., 72657.}	14.7624	0.0699161
27	newly 3 weeks spec	9364.	10784.	{10264., 11261.}	15.164	0.0610151	newly 3 weeks cumul	24919.	28503.	{27025., 29761.}	14.3825	0.0580415	newly 5 weeks spec	8776.	8649.13	{8217., 9162.}	3.17582	0.0139671	newly 5 weeks cumul	43318.	47362.3	{44952., 49482.}	9.33626	0.0385031	newly 56 weeks cumul	79621.	71514.3	{66758., 78583.}	10.3542	0.0480189
28	newly 3 weeks spec	11755.	13424.7	{13098., 13799.}	14.3073	0.0579494	newly 3 weeks cumul	27332.	31901.1	{30667., 32875.}	16.7169	0.0669822	newly 5 weeks spec	11093.	8500.08	{8217., 8938.}	23.3744	0.116169	newly 5 weeks cumul	50746.	52282.1	{51517., 53083.}	3.45324	0.0147946	newly 60 weeks cumul	87654.	64438.6	{63905., 65274.}	26.4852	0.133788
29	newly 3 weeks spec	8104.	9234.26	{8774., 9492.}	14.017	0.0567938	newly 3 weeks cumul	20099.	22384.6	{20944., 22944.}	11.4504	0.0468885	newly 5 weeks spec	8778.	9520.23	{9201., 9705.}	8.47448	0.0352381	newly 5 weeks cumul	37699.	41782.7	{39686., 42604.}	10.8894	0.0447582	newly 54 weeks cumul	75236.	80754.9	{75013., 81833.}	7.48639	0.031228
30	newly 3 weeks spec	13462.	17841.9	{16256., 18480.}	32.535	0.121703	newly 3 weeks cumul	25138.	31994.	{28783., 33459.}	27.2734	0.103504	newly 5 weeks spec	14527.	15051.7	{14777., 15579.}	6.05328	0.0263292	newly 5 weeks cumul	55394.	66322.7	{62159., 67781.}	19.729	0.0780165	newly 63 weeks cumul	91285.	90805.7	{81839., 92597.}	2.58305	0.0115605
31	newly 3 weeks spec	13467.	17110.3	{12867., 20739.}	30.9249	0.114864	newly 3 weeks cumul	21982.	28175.8	{19865., 35200.}	34.3085	0.126507	newly 5 weeks spec	17282.	15429.6	{12623., 18194.}	13.486	0.0654728	newly 5 weeks cumul	58272.	63720.8	{57489., 71939.}	11.0661	0.0447233	newly 64 weeks cumul	87865.	82940.6	{75096., 91330.}	8.52018	0.0392453
32	newly 3 weeks spec	11691.	12131.	{10504., 14438.}	11.6996	0.0483768	newly 3 weeks cumul	23586.	24336.1	{21286., 28491.}	10.2689	0.042922	newly 5 weeks spec	12032.	11062.1	{9160., 13904.}	18.1805	0.0844157	newly 5 weeks cumul	48919.	48660.2	{42789., 58577.}	12.8826	0.0555617	newly 61 weeks cumul	74173.	68146.6	{56965., 91129.}	20.0424	0.0929261
33	newly 3 weeks spec	9366.	9055.76	{8484., 9456.}	4.46018	0.0198468	newly 3 weeks cumul	20595.	20908.4	{19696., 22044.}	3.77514	0.0161498	newly 5 weeks spec	10722.	7389.41	{7005., 7655.}	31.3991	0.164256	newly 5 weeks cumul	42102.	37338.3	{35751., 38558.}	11.8744	0.0548812	newly 59 weeks cumul	87634.	50352.8	{48988., 50651.}	42.5419	0.241823
34	newly 3 weeks spec	6522.	4616.44	{4254., 5008.}	29.2174	0.150886	newly 3 weeks cumul	16514.	13566.4	{12570., 14473.}	17.8489	0.0859785	newly 5 weeks spec	7135.	3157.8	{2858., 3435.}	55.7421	0.355046	newly 5 weeks cumul	30872.	20737.3	{19462., 21995.}	32.8283	0.173261	newly 54 weeks cumul	67088.	27647.1	{26413., 28841.}	58.7898	0.385239
35	newly 3 weeks spec	10120.	10215.2	{10003., 10777.}	6.1586	0.0304436	newly 3 weeks cumul	20852.	20556.	{20074., 21601.}	4.24942	0.0214646	newly 5 weeks spec	12158.	12409.6	{12510., 13012.}	7.75835	0.0391138	newly 5 weeks cumul	45344.	45444.	{45566., 47243.}	5.36847	0.0273128	newly 59 weeks cumul	90553.	82587.7	{83109., 86571.}	8.8193	0.0469632
36	newly 3 weeks spec	8518.	5196.44	{4716., 5663.}	38.9946	0.215496	newly 3 weeks cumul	20967.	16333.2	{15209., 17351.}	22.1007	0.108959	newly 5 weeks spec	8858.	2635.06	{2355., 2940.}	70.2522	0.52834	newly 5 weeks cumul	39044.	22905.8	{21401., 24113.}	41.3333	0.232007	newly 57 weeks cumul	75101.	26809.3	{25400., 27973.}	64.3024	0.447664
37	newly 3 weeks spec	14436.	16162.6	{14287., 17154.}	12.7326	0.0513923	newly 3 weeks cumul	27190.	30742.4	{26288., 33176.}	14.2993	0.0572028	newly 5 weeks spec	14344.	13996.4	{13353., 14665.}	3.32982	0.014743	newly 5 weeks cumul	57920.	62150.9	{56585., 64725.}	8.32963	0.0345211	newly 62 weeks cumul	85971.	85902.2	{77091., 90350.}	4.63174	0.0205144
38	newly 3 weeks spec	14671.	6426.3	{3652., 9238.}	56.1973	0.384719	newly 3 weeks cumul	23153.	10905.	{6223., 15428.}	52.9003	0.351921	newly 5 weeks spec	16782.	12902.2	{9231., 16003.}	23.1189	0.124478	newly 5 weeks cumul	59731.	34277.7	{22047., 45781.}	42.6133	0.257549	newly 62 weeks cumul	80717.	72840.2	{55744., 92864.}	15.6423	0.0757692
39	newly 3 weeks spec	8107.	9401.62	{9106., 9696.}	16.0262	0.064395	newly 3 weeks cumul	18981.	21609.2	{21019., 22181.}	13.893	0.0563664	newly 5 weeks spec	8590.	9799.15	{9537., 10057.}	14.0763	0.0570005	newly 5 weeks cumul	36334.	41682.7	{40702., 42742.}	14.721	0.059514	newly 56 weeks cumul	64192.	72902.5	{70927., 71959.}	13.5694	0.0544386
40	newly 3 weeks spec	9375.	5193.3	{4745., 5607.}	44.6048	0.257392	newly 3 weeks cumul	23924.	16347.4	{14953., 17382.}	31.6693	0.166114	newly 5 weeks spec	8935.	2673.62	{2356., 2955.}	70.077	0.525363	newly 5 weeks cumul	42535.	22978.4	{21510., 24110.}	45.9776	0.267866	newly 57 weeks cumul	74746.	26919.7	{25779., 27912.}	63.9851	0.443738
41	newly 3 weeks spec	9002.	11842.1	{10528., 13034.}	31.5499	0.117411	newly 3 weeks cumul	21615.	28148.7	{24898., 30761.}	30.2278	0.11314	newly 5 weeks spec	8585.	10494.6	{10029., 10865.}	22.243	0.08698	newly 5 weeks cumul	39635.	50632.6	{46239., 54288.}	27.7472	0.105363	newly 58 weeks cumul	64657.	80140.6	{79259., 85032.}	23.9473	0.0926602
42	newly 3 weeks spec	13145.	16904.7	{16372., 17430.}	28.6016	0.108797	newly 3 weeks cumul	25907.	32666.9	{31186., 33607.}	26.123	0.100017	newly 5 weeks spec	11817.	13912.8	{13580., 14274.}	17.735	0.0707389	newly 5 weeks cumul	52038.	64214.6	{63226., 65113.}	23.3995	0.091121	newly 62 weeks cumul	70332.	87957.1	{87791., 88382.}	25.0598	0.0970334
43	newly 3 weeks spec	10255.	8727.56	{8413., 9033.}	14.8946	0.0703827	newly 3 weeks cumul	22579.	19841.4	{19341., 20523.}	12.1244	0.0565812	newly 5 weeks spec	11209.	10063.1	{9889., 10251.}	10.2232	0.0468806	newly 5 weeks cumul	45260.	39870.8	{39123., 40834.}	11.9072	0.055244	newly 55 weeks cumul	87509.	83450.9	{80185., 87620.}	4.69811	0.02118
44	newly 3 weeks spec	15502.	16487.7	{16261., 16874.}	7.26448	0.0306038	newly 3 weeks cumul	33206.	35931.1	{35361., 36963.}	9.21261	0.0384669	newly 5 weeks spec	12451.	11525.2	{11230., 11764.}	7.72532	0.0349862	newly 5 weeks cumul	61309.	62908.6	{62519., 63696.}	3.22207	0.0138403	newly 61 weeks cumul	87960.	81575.4	{80944., 81502.}	7.36694	0.0332828
45	newly 3 weeks spec	16177.	11612.3	{10645., 12315.}	28.2172	0.14598	newly 3 weeks cumul	31598.	22838.7	{20266., 24371.}	27.7211	0.142946	newly 5 weeks spec	13010.	13235.7	{13089., 13757.}	5.97087	0.0272939	newly 5 weeks cumul	61499.	50010.6	{47836., 52451.}	18.6806	0.0915752	newly 60 weeks cumul	80329.	84619.1	{81734., 90230.}	10.0455	0.0441526
46	newly 3 weeks spec	10769.	9532.88	{6934., 11060.}	13.1594	0.0681815	newly 3 weeks cumul	20202.	18458.5	{13805., 21158.}	12.1732	0.0607421	newly 5 weeks spec	14108.	12145.6	{8815., 13936.}	13.9428	0.0737565	newly 5 weeks cumul	48516.	42690.2	{31745., 48754.}	12.4951	0.0650178	newly 60 weeks cumul	82843.	75427.6	{52090., 87480.}	16.0354	0.0826153
47	newly 3 weeks spec	12425.	16662.	{14947., 17239.}	34.1004	0.126777	newly 3 weeks cumul	24055.	32000.1	{28221., 33502.}	33.0289	0.123064	newly 5 weeks spec	13477.	13893.5	{13549., 14198.}	3.3276	0.0141787	newly 5 weeks cumul	52391.	63487.9	{58097., 65124.}	21.181	0.0830824	newly 62 weeks cumul	79732.	86785.3	{77479., 88189.}	9.55228	0.0395646
48	newly 3 weeks spec	12536.	8524.6	{6441., 10030.}	33.5152	0.185317	newly 3 weeks cumul	21158.	14608.4	{10956., 16824.}	33.243	0.181845	newly 5 weeks spec	16548.	14221.9	{11575., 16194.}	14.0568	0.0700416	newly 5 weeks cumul	54873.	41671.7	{33957., 47701.}	24.7612	0.128899	newly 62 weeks cumul	90499.	77455.9	{58942., 92992.}	15.8592	0.0810579
49	newly 3 weeks spec	21183.	16486.8	{12518., 20550.}	22.2127	0.118872	newly 3 weeks cumul	34718.	26899.8	{19139., 34672.}	22.798	0.125386	newly 5 weeks spec	14957.	15560.3	{13409., 18281.}	10.8837	0.0455924	newly 5 weeks cumul	71735.	62450.3	{56915., 71633.}	12.998	0.0626846	newly 64 weeks cumul	87984.	82058.	{75119., 91323.}	8.94671	0.0411949
50	newly 3 weeks spec	10269.	10827.2	{10659., 11029.}	5.5614	0.0234608	newly 3 weeks cumul	26490.	27825.	{27404., 28285.}	5.11231	0.0216217	newly 5 weeks spec	9271.	9500.07	{9412., 9673.}	3.37644	0.0145758	newly 5 weeks cumul	46170.	47976.1	{47668., 48467.}	4.22723	0.0179947	newly 55 weeks cumul	81778.	82149.4	{82487., 83151.}	1.98715	0.00894692
